# Lignin-Acrylic Acid Copolymer as an Effective Emulsifier for Oil-Water Emulsion

**DOI:** 10.3390/polym18091056

**Published:** 2026-04-27

**Authors:** Shirin Fatehi, Pedram Fatehi, Ehsan Behzadfar, Leila Pakzad

**Affiliations:** 1 Department of Chemical Engineering, Lakehead University, Thunder Bay, ON P7B 5E1, Canada; sfatehi@lakeheadu.ca; 2 Sustainable Polymers Research Lab (SPRL), Center for Packaging Innovation and Sustainability (CPIS), The Creative School, Toronto Metropolitan University, Toronto, ON M5B 2K3, Canada; behzadfar@torontomu.ca

**Keywords:** lignin, dispersion, emulsion, adsorption, rheology

## Abstract

Oil–water emulsions constitute essential components in a wide range of industries. Despite their extensive use in emulsion systems, synthetic emulsifiers are often associated with environmental concerns and high costs. In this study, lignin—a by-product of the pulping industry—was polymerized with acrylic acid and employed as an emulsifier in a xylene–water system to address this challenge. When testing two lignin–acrylic acid copolymers, the results confirmed that the one possessing a higher molecular weight (7.99 × 10^5^ g/mol) and charge density (4.7 mmol/g) (KL-AA-10) generated xylene–water emulsions with improved stability, and higher viscosity and viscoelastic moduli. These observations were consistent with the greater adsorption of this polymer, relative to the counterparts with a lower molecular weight and charge density at the xylene–water interface, as monitored using a Quartz Crystal Microbalance. The adsorption of KL-AA-10 resulted in the formation of smaller emulsion droplets (D50 = 0.6 µm) within the system, as evidenced by confocal microscopy analysis. This study underscores the potential of lignin as a renewable emulsifier for diverse applications.

## 1. Introduction

Oil–water emulsions are widely applied across industries, including food, cosmetics, pharmaceuticals, and petrochemicals [1,2]. Despite their extensive use, emulsions are thermodynamically unstable and require emulsifiers to achieve kinetic stability [2]. At present, synthetic emulsifiers are employed in oil–water emulsions; however, their use is increasingly discouraged due to the environmental concerns related to their production and application. To address these challenges, biobased emulsifiers—such as cellulose and chitosan—have been developed and investigated as alternatives for stabilizing oil–water emulsions [3,4,5,6,7,8]. Although notable advances have been achieved in this area, there remains a need to develop new, effective bio-based emulsifiers for diverse emulsion systems.

Lignin is a heterogeneous macromolecule and represents the largest natural reservoir of aromatic compounds on Earth [3]. Despite its abundance in lignocellulosic resources and the existence of established industrial processes for its commercial production [9], its utilization in value-added applications remains largely limited to energy generation [10]. Consequently, there is strong motivation to identify high-value applications for lignin. Chemical modification methods—including oxidation, sulfoalkylation, and carboxyalkylation—have been widely utilized to produce water-soluble lignin derivatives, which have been used as dispersants in suspension systems [11] and as emulsifiers in emulsion systems [12,13]. Although promising results have been reported for sulfonated lignin as an emulsifier, knowledge regarding the production and application of lignin-derived emulsifiers remains limited. Previously, lignin–acrylic acid copolymers demonstrated strong performance as flocculants for suspension systems [14]. However, its utilization as an emulsifier has not been assessed yet. The objective of the present work was to assess their emulsifying performance and thereby expand understanding of lignin-derived emulsifier applications.

The preparation of stable emulsions requires careful control of interfacial characteristics [15]. Emulsifiers typically adsorb onto droplet surfaces within emulsion systems, thereby modifying interfacial properties [16,17]. Recent studies have demonstrated that interfacial performance in aqueous systems is governed not only by amphiphilicity but also by surface chemistry, charge density, and molecular architecture. In particular, increasing charge density enhanced electrostatic interactions and colloidal stability [7,18]. Molecular weight also played a critical role in emulsion stabilization. For example, lower-molecular-weight (90 kDa) hydroxypropyl cellulose (HPC) polymers were unable to stabilize palm and sunflower oil emulsions, whereas stable emulsions with small droplets formed when high-molecular-weight (≳400 kDa) HPC polymers were used due to sufficient viscosity of the continuous phase to hamper creaming and long-range steric repulsion to prevent droplet coalescence [19]. In another study, the octenyl succinic acid sodium starch/chitosan complexes with a high-molecular-weight exhibited accelerated interfacial diffusion and rearrangement, leading to a rapid reduction in interfacial tension [20]. These factors collectively determine adsorption kinetics, interfacial viscoelasticity, and ultimately system stability.

To develop effective lignin-derived emulsifiers, it is essential to understand the relationship between the physicochemical characteristics of lignin-based polymers and their emulsifying performance. In this regard, the degree of oxidation has been shown to influence the performance of oxidized lignin as a coagulant for dye solutions and as a dispersant in cement admixtures [21,22]. Additionally, the effects of charge density and molecular weight on the flocculation performance of lignin polymers in aluminum oxide suspensions have been documented [10,23,24]. Given that lignin–acrylic acid copolymers can be synthesized with varying molecular weights and charge densities [14], it is necessary to determine how these parameters influence their emulsifying performance in oil–water systems.

While sulfonated and oxidized lignin derivatives have been widely explored as bio-based emulsifiers, the modification mainly introduces small functional groups (e.g., sulfonate or carboxyl groups) onto the lignin backbone. These modifications primarily increase lignin hydrophilicity and rely mainly on electrostatic stabilization mechanisms. In contrast, lignin–acrylic acid copolymer is expected to significantly alter the interfacial behavior of lignin by increasing the density of carboxylic acid groups, leading to stronger electrostatic repulsion [25]. The increase in molecular weight through polymer grafting will also enhance steric stabilization via extended polymer chains at the interface [26]. Thus, the novelty of this work lies in the design of lignin–acrylic acid (KL-AA) copolymers that simultaneously increase charge density and molecular weight through graft polymerization, enabling combined electrostatic and steric stabilization mechanisms that are not achievable with conventional lignin modifications. Such structural features are expected to influence adsorption behavior, interfacial layer viscoelasticity, and ultimately emulsion stability. Xylene was selected as the oil phase to model a synthetic oil–water emulsion system because it is a commonly used model aromatic solvent in emulsion studies due to its well-defined composition, moderate viscosity, and relatively low volatility compared with lighter hydrocarbons [27]. These characteristics allow the reproducible evaluation of interfacial stabilization behavior without the compositional complexity associated with crude oil or fuel mixtures. The adsorption behavior of lignin–acrylic acid copolymers at the xylene interface was monitored through dynamic interfacial tension measurements. Furthermore, the effects of polymer adsorption on interfacial tension, rheological properties, and the stability of xylene–water emulsions were systematically investigated. This study aims to advance the application of lignin as a renewable, biobased emulsifier for industrial use.

## 2. Experimental Methodology

### 2.1. Materials

Softwood kraft lignin (KL) (sulfur content: 2.0 wt.%; Mw: 2.13 × 10^5^ g/mol; Mw/Mn: 1.58), produced via the LignoForce^TM^ technology, was received from FPInnovations. Acrylic acid (AA), potassium persulfate (K_2_S_2_O_8_), sodium hydroxide (NaOH, 97%), sulfuric acid (H_2_SO_4_, 98%), dimethyl sulfoxide-d_6_ (DMSO-d_6_), deuterium oxide (D_2_O), acrylic acid (AA), polydiallyldimethyl-ammonium chloride (PDADMAC, 100,000–200,000 g/mol), potassium hydroxide (0.8 N), para-hydroxy benzoic acid (0.5%), KCL (1 M) and cellulose membrane tubes (1000 g/mol cut off) were purchased from Sigma-Aldrich (Oakville, ON, Canada). Xylene (C_6_H_4_(CH_3_)_2_ ≥ 98.5%, ACS grade as a mixture of ortho, meta, and para isomers) was purchased from Fisher Scientific (Waltham, MA, USA)**.** All chemicals were used without further purification. HPLC-grade water produced by a Milli-Q water purifier was used in the QCM and zeta potential experiments.

### 2.2. Polymerization of Kraft Lignin and Acrylic Acid

The free-radical polymerization of lignin with acrylic acid (AA) was conducted in 100 mL three-neck flasks under a nitrogen atmosphere, following a previously established procedure [14]. Initially, 4 g of lignin were dispersed in 20 mL of deionized water at room temperature and stirred at 300 rpm for 1 h to ensure uniform suspension. While maintaining stirring, predetermined amounts of AA were added to the flasks. After adjusting the pH to 3, the total reaction volume was adjusted to 40 mL using deionized water. Following deaeration, the reaction mixture was transferred to a preheated water bath maintained at 80 °C. After 5 min of equilibration, a preheated (80 °C) initiator solution of potassium persulfate (K_2_S_2_O_8_, 1.5 wt.%) was introduced to initiate the polymerization under continuous stirring at 300 rpm. The reaction proceeded for 3 h, after which it was quenched. The pH was adjusted to 1.5 by stirring the reaction medium for 30 min to facilitate precipitation of the lignin–acrylic acid (KL-AA) copolymer [14]. Subsequently, the reaction mixture was centrifuged at 4000 rpm for 10 min to remove the remaining poly(acrylic acid) (PAA) homopolymer and unreacted AA monomer in the supernatant and to collect the precipitated KL-AA polymer. The recovered product was then resuspended in water, and its pH was adjusted to 7 prior to membrane dialysis for 2 days to remove residual impurities and unreacted monomers. Finally, the purified samples were dried in an oven at 105 °C [14].

The polymerization process was repeated using two different AA-to-lignin molar ratios (3.5 and 10), and the resulting products were designated as KL-AA-3.5 and KL-AA-10, respectively.

### 2.3. H-NMR Analysis

The chemical structures of KL and KL-AA polymers were characterized using ^1^H-NMR spectroscopy. In these experiments, 70 mg of KL was dissolved in 1 mL of dimethyl sulfoxide (DMSO), while 70 mg of KL-AA was dissolved in 1 mL of deuterium oxide (D_2_O). Each solution was stirred overnight to ensure complete dissolution of the lignin derivatives. The ^1^H-NMR spectra were recorded using a nuclear magnetic resonance spectrometer (AVANCE NEO-1.2 GHz, Bruker Corporation, Billerica, MA, USA). Measurements were conducted with 64 scans, employing a 90° pulse and a relaxation delay of 1.00 s. Due to the different solubility characteristics of KL and KL-AA, different deuterated solvents were used for NMR measurements to obtain spectra with sufficient signal quality. It should be noted that the solvent may introduce slight variations in chemical shift positions in the NMR analysis [28]. Therefore, the spectra were primarily interpreted based on the presence of characteristic functional group signals rather than direct comparison of peak positions.

### 2.4. Solubility, Charge Density, and Carboxylic Acid Group Analyses

Initially, a 1 wt.% solution of lignin samples was prepared by mixing the lignin with deionized water at pH 7 and room temperature. The mixtures were shaken for 1 h at 100 rpm and 30 °C to facilitate dissolution. Subsequently, the solutions were centrifuged at 1000 rpm for 5 min to separate the soluble fractions of the lignin derivatives from the insoluble residues. In one set of experiments, the insoluble lignin derivatives retained on the filter after filtration were dried and weighed to determine their mass. This measurement enabled the calculation of lignin solubility through a mass balance approach. The filtrate was then collected for charge density analysis of the soluble lignin fraction. The charge density of the lignin samples was measured using a Particle Charge Detector (Mutek PCD-04 titrator, Herrsching, Germany) with standard solutions of PDADMAC or PVSK (~0.005 mol/L), following the standard TAPPI T 651 procedure. The titration was conducted at room temperature, and the sample solutions were adjusted to pH 7.0 prior to analysis.

The contents of carboxylic acid groups and phenolic hydroxyl groups in KL and KL-AA polymers were determined using an automatic potentiometric titrator (785 DMP Titrino, Metrohm, Herisau, Switzerland). Approximately 0.06 g of dried KL or KL-AA polymer was added to 100 mL of deionized water containing 1 mL of 0.8 mol/L potassium hydroxide in a 250 mL beaker. The mixture was stirred at 200 rpm for 5 min, after which 4 mL of a 0.5% para-hydroxybenzoic acid solution was added as an internal standard to improve the identification of inflection points in the potentiometric titration curve of lignin polymers. Such internal standardization has been previously used in lignin titration methods to resolve overlapping acid functionalities and improve quantification accuracy [29,30]. The solution was then titrated with 0.1 mol/L hydrochloric acid. During titration, three sequential endpoints (V′_1_, V′_2_, and V′_3_) appeared as the pH decreased. The corresponding endpoints for the blank sample (i.e., without lignin) were recorded as V_1_, V_2_, and V_3_, respectively. The carboxylic acid and phenolic hydroxyl contents of the samples were subsequently calculated according to Equations (1) and (2) [14].

(1) $$\mathrm{Phenolic}\;\mathrm{hydroxyl}\;\mathrm{group}\;\left(\frac{mmol}{g}\right)=\frac{C_{HCl}\;\left[{(V}_{2}^{\prime }-V_{1}^{\prime }\right)-(V_{2}-V_{1})]}{m}$$

(2) $$\mathrm{Carboxylic}\;\mathrm{acid}\;\mathrm{group}\;\left(\frac{mmol}{g}\right)=\frac{\;C_{HCl}\;\left[{(V}_{3}^{\prime }-V_{2}^{\prime }\right)-(V_{3}-V_{2})]}{m}$$

where C_HCl_ is the concentration of HCl solution (0.1 mmol/L) as the titrant, m is the mass (g) of the sample. In these equations, V_1_, V_2_, and V_3_ were the volumes (mL) of HCl solution consumed for the three endpoints in blank titration, and V′_1_, V′_2_, and V′_3_ were the volumes (mL) of HCl solution consumed for the three endpoints in sample titration, respectively. Results are the average of three repetitions.

The grafting ratio was calculated based on the amount of carboxyl group according to Equation (3) [31].

(3) $$Grafting\;ratio\;\left(\%\right)=\frac{100\times C\times 94}{1-C\times 94}$$

where *C* is the concentration of carboxyl group in KL-AA, mol/g. 94 g/mol is the molecular weight of the sodium form of acrylic acid.

### 2.5. Molecular Weight Analysis

The molecular weight of the samples was determined using gel permeation chromatography (GPC) equipped with multi-detectors (Malvern, Worcestershire, UK, model GPCmax VE2001 Module coupled with a Viscotek TDA305 system, including UV, refractive index (RI), viscometer, low-angle, and right-angle laser detectors). For KL analysis, PAS106M, PAS103, and PAS102.5 columns were employed. Tetrahydrofuran (THF) was used as the eluent at a constant flow rate of 1.0 mL/min, and polystyrene served as the calibration standard. For KL-AA polymer analysis, PAA206 and PAA203 columns were used. A sodium nitrate (NaNO_3_) solution (50 g/L) was employed as the eluent at a fixed concentration of 0.1 mol/L, and polyethylene oxide was used as the calibration standard. All measurements were conducted in triplicate, and the reported values represent the averages of three independent determinations. It should be noted that the molecular weights reported here are apparent values obtained from calibration with linear polymer standards. Because lignin possesses a highly branched and heterogeneous structure, deviations from absolute molecular weight values may occur. In addition, different eluent systems and calibration standards were used for KL and KL-AA polymers due to differences in their solubility and polymer composition. Therefore, the GPC results were mainly intended for relative comparison and to confirm the increase in molecular weight after graft polymerization.

### 2.6. Surface Tension and Critical Aggregation Concentration

For surface tension measurements, KL-AA solutions (25 g/L) were diluted to prepare lignin-containing test solutions at the desired concentrations. The surface tension of these solutions was measured at room temperature using the Du Noüy ring method with a tensiometer (Sigma 701, Biolin Scientific, Gothenburg, Sweden) operated through OneAttension software (version 4.0). Prior to each measurement, the small vessel (30 mL capacity) was thoroughly rinsed with deionized water, and the Du Noüy ring was flame-annealed and rinsed sequentially with ethanol and ultrapure water to eliminate any organic residues prior to each measurement, minimizing contamination-related errors. Subsequently, 20 mL of the lignin solution was transferred into the vessel, and surface tension measurements were recorded at 10 points for each sample. The resulting data points were used to determine the critical aggregation concentration (CAC) of the lignin samples. Reported values represent the averages of 10 independent measurements.

### 2.7. Dynamic Interfacial Tension

The dynamic interfacial tension (γ) between KL-AA solutions at different concentrations and the organic phase was measured using an Attension Theta Biolin optical tensiometer (Gothenburg, Sweden), employing the pendant drop method [32]. In each experiment, 3 mL of xylene was transferred into a quartz cuvette, which was then sealed to minimize solvent evaporation. A 5 µL droplet of KL-AA aqueous solution (20 g/L) was generated at the tip of a needle and carefully introduced into the oil phase [33]. Droplet images were recorded over a total duration of 3600 s to allow sufficient time for adsorption equilibrium to be approached [34], with a capture rate of 10 images per second during the first 600 s and 1 image per minute for the remaining period. The interfacial tension values were calculated by analyzing the droplet shape based on the Young–Laplace equation [35]. The aqueous phase was used as the pendant droplet to allow diffusion of KL-AA molecules to the oil–water interface [33,36]. It should be noted that equilibrium interfacial tension is independent of droplet configuration, although adsorption kinetics may differ from those in oil-in-water emulsions [37,38]. A slight difference can be observed between the pendant drop configurations of water drop and oil drop [37]. The measurements are therefore interpreted as comparative indicators of interfacial activity rather than the exact representations of emulsification environments. All measurements were conducted in triplicate, and representative curves are shown due to the continuous nature of the data.

### 2.8. Zeta Potential

The zeta potential of KL-AA solutions was measured using a NanoBrook PALS analyzer (Brookhaven Instruments Corporation, Holtsville, NY, USA). KL-AA stock solutions (20 g/L) were prepared by stirring at 300 rpm and 25 °C overnight to ensure complete dissolution. Separately, an electrolyte solution was prepared by adding 250 μL of potassium chloride (KCl) to 1 L of Milli-Q water, followed by stirring for 30 min. Subsequently, 20 mL of the KCl solution was filtered and used for the measurements. For analysis, 50 μL of the KL-AA solution was added to 50 mL of the filtered KCl solution and sonicated for 15 s to ensure uniform dispersion. Zeta potential measurements were conducted at room temperature under a constant electric field strength of 8.4 V/cm. The reported values represent the averages of three independent measurements.

### 2.9. Adsorption Analysis

The adsorption behavior of lignin derivatives (5 g/L) was evaluated using xylene-coated aluminum oxide sensors (QSX-309). These sensors were selected as a solid support for forming a xylene-coated surface, enabling the investigation of KL-AA adsorption dissolved in water at a model hydrophobic interface of xylene. Xylene is a nonpolar and electrically neutral solvent that serves as a hydrophobic phase that mimics the oil environment. Prior to coating, the sensors were cleaned by sonication in 99 vol% ethanol for 15 min. The cleaned sensors were then dried under a nitrogen stream and subsequently treated with UV/ozone (PSD Series digital UV ozone system, Novascan Technologies, Ames, IA, USA) for 15 min to remove residual contaminants. Xylene coating was performed by depositing 5 µL of xylene onto the sensor surface, followed by spin-coating at 3000 rpm for 30 s under a nitrogen atmosphere, with an acceleration rate of 200 m/s^2^ [12]. After coating, the sensors were dried in an oven at 110 °C for 30 min. This coating and drying procedure was repeated for 10 consecutive cycles to ensure uniform and stable xylene coverage. Although xylene is a volatile solvent, repeated spin-coating cycles promote the adsorption of hydrocarbon molecules onto the aluminum oxide surface, resulting in a thin hydrophobic organic layer after solvent evaporation. The coated sensors exhibited stable QCM-D baselines prior to adsorption measurements, indicating that the surface layer remained intact during the experiments.

Adsorption measurements were conducted using a Quartz Crystal Microbalance with Dissipation monitoring (QCM-D 401, E1, Q-Sense Inc., Gothenburg, Sweden). Initially, the coated sensors were rinsed with a buffer solution (Milli-Q water) until a stable baseline was achieved. The buffer solution was then replaced with KL-AA solutions (5 g/L), and adsorption experiments were performed at a flow rate of 0.15 mL/min and room temperature. Following 20 min of adsorption, the sensors were rinsed again with the buffer solution to remove loosely bound polymer chains and evaluate the stability of the adsorbed layer. It should be mentioned that a KL-AA concentration of 5 g/L was used in the QCM-D experiments to ensure stable adsorption measurements. Higher polymer concentrations can lead to excessive mass deposition and large dissipation responses, which complicate the interpretation of QCM-D data. Therefore, a lower concentration was selected to allow reliable monitoring of adsorption kinetics and comparison between samples.

### 2.10. Emulsion Preparation

Initially, KL-AA stock solutions (20 g/L) were prepared. These solutions were subsequently mixed with xylene at an equal volumetric ratio (i.e., xylene volume fraction of 50 vol%) in clean glass vials. The resulting mixtures were then emulsified using an ultrasonic homogenizer (Omni-Ruptor 4000, Omni International Inc., Kennesaw, GA, USA) equipped with a 9.5 mm diameter titanium probe tip at room temperature, operating at a power of 240 W for a total sonication time of 30 s, applied in 3 s intervals. Based on the sample volume (10 mL), the corresponding energy density was 720 J/mL.

Preliminary experiments with unmodified kraft lignin showed poor water solubility and an inability to form stable emulsions under the investigated conditions and, therefore, were not included in further analysis.

### 2.11. Rheological Properties

Emulsions containing lignin–acrylic acid (KL-AA) copolymers were prepared as described above. The rheological properties of the emulsions were measured at room temperature using a hybrid rheometer (TA Instruments, New Castle, DE, USA) equipped with a cone-and-plate geometry (cone diameter: 28.03 mm; cone length: 41.96 mm; cone angle: 1°; gap: 5500 μm). For each measurement, 1 mL of emulsion was carefully transferred onto the lower plate of the rheometer using a pipette. The viscosity of the samples was determined over a shear rate range of 0.1 to 100 s^−1^. In addition, amplitude sweep tests were conducted over a strain range of 0.1–100% at a constant angular frequency of 10 rad/s to evaluate the viscoelastic behavior of the emulsions. Furthermore, frequency sweep measurements were conducted in the range of 0.1 and 100 rad/s. Rheological measurements were performed in repeated runs, and highly consistent flow and viscoelastic responses were obtained; representative curves are presented for clarity.

### 2.12. Stability Analysis

In this set of experiments, 6 mL of KL-AA-included emulsions were prepared by mixing 2 mL of KL-AA-containing aqueous solution with 2 mL of xylene in glass vials. The emulsions were generated through ultrasonication at a power of 240 W for 30 s in 3 s intervals. Subsequently, the samples were further homogenized using a vortex mixer (VWR) at 2500 rpm for 10 s. Following preparation, the emulsions were transferred to a vertical scan analyzer (Turbiscan Lab Expert, Formulaction, Toulouse, France) and monitored at room temperature for 24 h to evaluate their stability. Stability measurements were repeated, and similar stability profiles were observed across runs, confirming the reproducibility of the results. Representative curves are presented due to the continuous nature of the data.

### 2.13. Visual Analysis

The microstructure of the emulsions was analyzed immediately after preparation using a Leica TCS SP8 confocal laser scanning microscope (Leica Microsystems Inc., Wetzlar, Germany) equipped with a white light laser (WLL) operating at an excitation wavelength of 563 nm. Imaging was performed using an HC PL APO CS2 100×/1.40 oil immersion objective lens. For sample preparation, 40 µL of the emulsion was stained with 5 µL of an aqueous Nile Red dye suspension (0.5 wt.%). Nile Red was used as a fluorescent probe to visualize the oil phase. As a polarity-sensitive dye, Nile Red exhibits strong fluorescence in nonpolar environments and weak fluorescence in aqueous media. Therefore, the dye preferentially partitions into the oil droplets, enabling the visualization of droplet morphology in the emulsion. The stained samples were then placed onto glass slides and covered with a cover slip. Red fluorescence was used to visualize the oil phase, and images were acquired using a 600–710 nm emission filter under 563 nm laser illumination. Also, the ImageJ software (version 1.54s) was used to quantify the droplet size of the emulsions.

## 3. Results and Discussion

### 3.1. Lignin-Acrylic Acid Properties

In this work, the copolymerization of kraft lignin (KL) and acrylic acid (AA) was carried out under previously optimized conditions (i.e., 15 g/L potassium persulfate, 80 °C, and 3 h) [14]. The physicochemical properties of the resulting KL-AA copolymers are summarized in Table 1. The lignin samples synthesized at a higher AA-to-lignin molar ratio exhibited increased molecular weight (7.99 × 10^5^ g/mol vs. 3.07 × 10^5^ g/mol), charge density (4.7 mmol/g vs. 1.1 mmol/g), and carboxylic acid group content (3.23 mmol/g vs. 1.09 mmol/g). The grafting ratio was calculated to be 11.4% and 43.6% for KL-AA-3.5 and KL-AA-10, respectively. While our study monitored overall grafting ratio and functional group changes, it should be noted that KL is inherently polydisperse, and its polymerization may selectively modify certain subunits, leading to compositional drift. This heterogeneity could contribute to variability in the final material properties. These increases are attributed to enhanced copolymerization efficiency between lignin and AA at higher AA-to-lignin ratios [39]. Consistent with these findings, the KL-AA-10 solution (20 g/L) displayed a slightly higher zeta potential (ζ) compared to KL-AA-3.5. The more negative ζ value of KL-AA-10 is associated with its higher carboxylate group content (Table 1), as carboxyl groups are deprotonated under neutral pH conditions [40]. The phenolic hydroxyl content of KL, originally measured at 3.26 mmol/g, decreased to 1.58 and 0.84 mmol/g following polymerization with AA at molar ratios of 3.5 and 10 (AA/lignin), respectively (Table 1).

The copolymerization mechanism of KL with AA under acidic conditions is primarily driven by initiator decomposition [14]. Specifically, the thermal self-decomposition of potassium persulfate generates sulfate radicals [41], which abstract hydrogen atoms from lignin hydroxyl groups, thereby forming lignin macroradicals. These macroradicals subsequently react with AA monomers, initiating chain propagation and polymer formation [42,43]. Polymerization with AA significantly enhanced the solubility of KL. Moreover, increased copolymerization of KL-AA resulted in reduced hydrogen and carbon contents (Table 1), which is consistent with the incorporation of carboxylic acid functionalities into the lignin structure [44].

### 3.2. Chemical Structure of Lignin Derivatives

**Figure 1:**
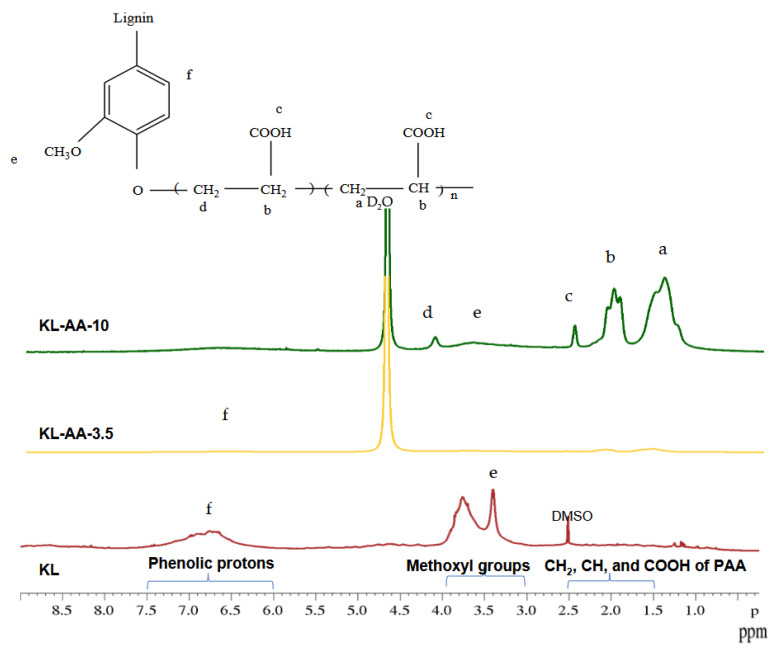
Chemical structure and ^1^H-NMR of lignin derivatives.

Figure 1 presents the ^1^H-NMR spectra of KL and KL-AA copolymers. The signals observed in the range of 6.0–7.5 ppm are attributed to aromatic protons associated with phenolic hydroxyl structures in lignin (label f in Figure 1). The peaks appearing at 3.0–3.9 ppm correspond to protons in methoxy groups, while those in the range of 1.4–2.4 ppm are assigned to protons of CH_2_, CH, and COOH groups in lignin–acrylic acid structures present in both KL-AA-3.5 and KL-AA-10. The signals detected at 4.5–4.9 ppm originate from the D_2_O solvent. Additional peak characteristics of the KL-AA copolymer chain segments are observed at 1.1 ppm, 1.9 ppm, and 2.4 ppm. Specifically, the signal at 1.1 ppm is attributed to C-1 protons (a), the peak at 1.9 ppm corresponds to C-2 protons (b), and the signal at 2.4 ppm is associated with the carboxylic acid protons of poly(acrylic acid) (c) [44]. Furthermore, the presence of KL-related peaks at 2.50 ppm, 3.2–3.9 ppm, and 6.5–7.1 ppm in the KL-AA spectra confirms the incorporation of lignin into the polymer structure, indicating successful polymerization between KL and AA. Similarly, characteristic KL signals observed at 3.20 ppm, 2.55–3.0 ppm, 5.15–5.75 ppm, 5.99–7.42 ppm, 8.30 ppm, and 9.2 ppm further support the formation of lignin–acrylic acid copolymers. In addition, the peak at approximately 4.10 ppm in the KL-AA spectra is assigned to –CH_2_– protons (d in Figure 1) linked to the aromatic ring through an ester bond (–CH_2_–O–C_6_H_5_). Notably, the KL-AA-10 sample exhibits more intense peaks associated with acrylic acid units, particularly in the 1.5–2.5 ppm region corresponding to –CH_2_–O–C_6_H_5_–n structures, reflecting the higher AA content in this copolymer.

### 3.3. Lignin Derivative Water and Oil Interactions

**Figure 2:**
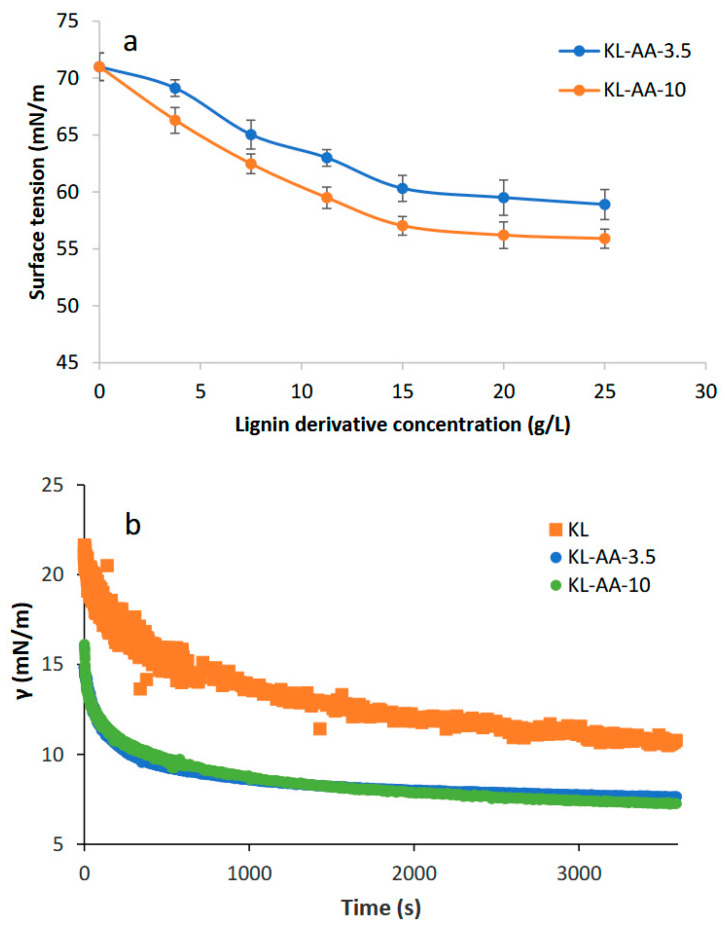
(**a**) Surface tension of lignin solution as a function of lignin concentration, (**b**) the interfacial tension of KL-AA samples in the xylene–water system.

The critical aggregation concentrations (CAC) of KL-AA-3.5 and KL-AA-10 were determined using the Du Noüy ring method by monitoring changes in the surface tension of lignin-containing solutions (Figure 2a). This analysis enabled the identification of the concentration at which lignin molecules begin to self-associate in solution. In a previous study on kraft lignin–tannic acid (KL-TA) systems, the surface tension of pure water was reported as 72.8 mN/m, which decreased to approximately 69 mN/m with increasing KL-TA concentration due to the spontaneous adsorption of lignin molecules at the air–water interface [45]. In the present work, the surface tension of pure water was measured as 71 mN/m. As the concentration increases upon the addition of KL-AA polymers, KL-AA molecules accumulate at the air–water interface, resulting in a gradual decrease in surface tension (Figure 2a). When the concentration approaches the CAC, the surface tension reaches a plateau, suggesting that additional KL-AA molecules preferentially form aggregates in the bulk solution. Furthermore, increasing the concentration of KL-AA resulted in a more pronounced reduction in surface tension for KL-AA-10 compared to KL-AA-3.5, which is attributed to its higher carboxylic acid group content and greater solubility in water (Table 1).

Although KL-AA-10 exhibited lower surface tension values compared with KL-AA-3.5 at all concentrations, the breakpoint in the γ–log C plots occurred at approximately the same concentration (~15 g/L), indicating that the CAC for both samples was approximately 15 g/L (Figure 2a). This suggests that KL-AA-10 has stronger interfacial activity but that the onset of aggregation in bulk solution occurs at comparable concentrations for both polymers. The relatively high CAC value to conventional surfactants can be attributed to the polymeric nature and structural heterogeneity of KL-AA, which require higher concentrations to achieve effective interfacial coverage and aggregation. The CAC of polymeric systems is often less sharply defined and occurs over a broader concentration range due to gradual association and adsorption processes, in contrast to the well-defined critical micelle concentration of small-molecule surfactants [46]. Similar behavior has been reported for lignin-based emulsifiers [18,47]. These findings suggest that below this concentration, KL-AA molecules primarily adsorb at the water surface. In contrast, at concentrations above the CAC (e.g., 20 g/L), the polymers not only saturate the interface but also form self-aggregates in the bulk solution. Since previous studies have reported enhanced emulsifying performance of lignin derivatives at concentrations exceeding their CAC [12], a concentration of 20 g/L was selected for subsequent experiments in this study.

To further evaluate the interfacial activity relevant to emulsification, Figure 2b illustrates the interfacial tension of KL-AA samples in the xylene–water system. As expected, the presence of KL-AA polymers reduced the interfacial tension (γ) of the mixture more effectively than unmodified KL, and the rate of reduction was more pronounced for the KL-AA samples. The reduction in xylene–water interfacial tension indicates that KL-AA molecules can effectively adsorb at the oil–water interface. This adsorption lowers the interfacial free energy and facilitates the formation and stabilization of oil–water emulsions. However, both KL-AA-3.5 and KL-AA-10 exhibited similar interfacial tension values at equilibrium, indicating that their adsorption at the oil–water interface reached a saturation state [32,48]. Because KL is water-insoluble and contains fewer carboxylic acid groups compared to KL-AA derivatives, these findings suggest that the presence of carboxylic acid functionalities—along with improved water solubility—play a critical role in lowering interfacial tension in the system. Owing to its poor solubility and limited effectiveness in reducing interfacial tension, the KL sample was excluded from subsequent experiments and analyses.

### 3.4. Insights into Adsorption Behavior of Lignin-Acrylic Acid on Xylene-Coated Surface

**Figure 3:**
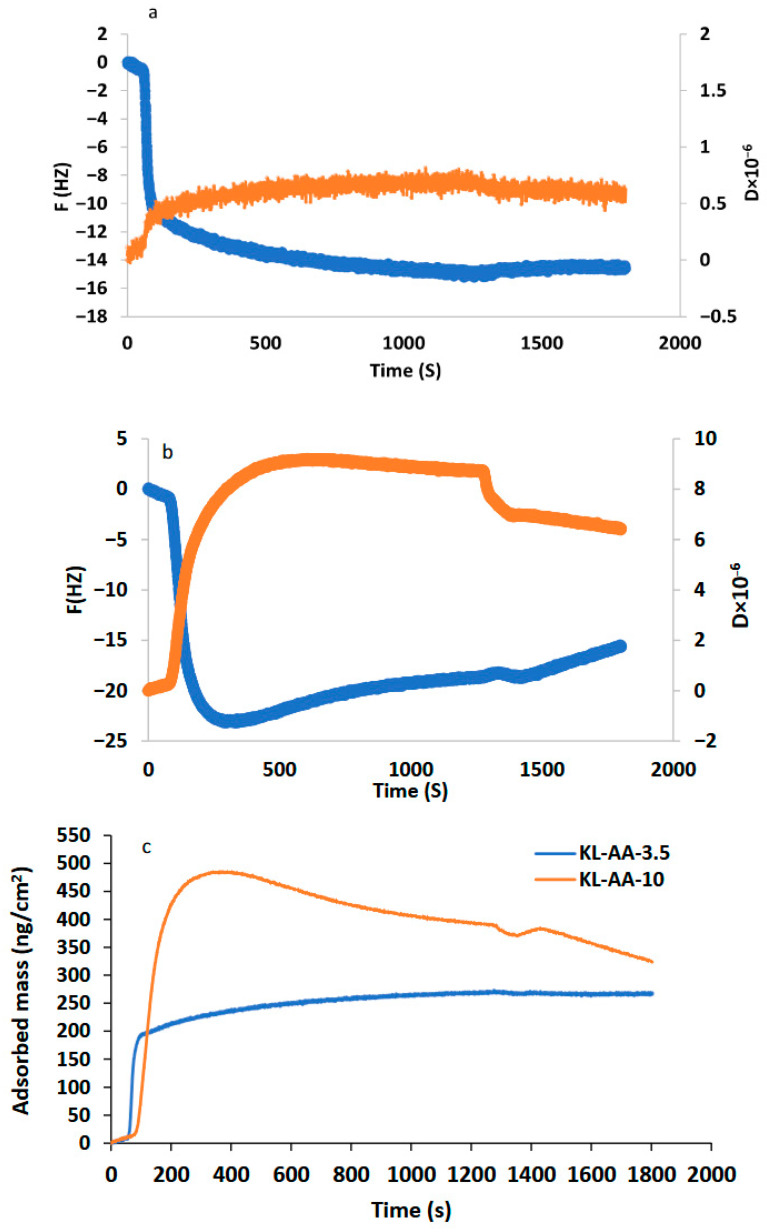
Frequency and dissipation of (**a**) KL-AA-3.5 and (**b**) KL-AA-10 (orange color line: D, blue color line: F) and (**c**) adsorbed mass of KL-AA-3.5 and KL-AA-10 on xylene-coated sensors as a function of time.

Xylene-coated Al_2_O_3_ sensors were employed to investigate the adsorption behavior of KL-AA-3.5 and KL-AA-10. Upon adsorption of KL-AA onto the coated sensors, a significant decrease in frequency accompanied by a noticeable increase in dissipation was observed, indicating mass deposition and the formation of a soft and hydrated adlayer—subsequent buffer rinsing led to the partial recovery of both frequency and dissipation signals. The relatively large dissipation change suggests that the adsorbed film exhibits viscoelastic characteristics rather than behaving as a rigid layer. In such systems, the viscoelastic Voigt model was typically used to describe the mechanical properties of the adsorption layer rather than the Sauerbrey equation (practical when a rigid layer forms). The KL-AA solutions were introduced following stabilization of the baseline through buffer rinsing. The deposition of these polymers onto the coated sensors resulted in measurable changes in frequency (Δf) and dissipation (ΔD). It adsorbed mass of KL-AA on the xylene-coated sensors, as presented in Figure 3. In both cases, KL-AA-3.5 exhibited a smaller change in dissipation (ΔD) compared to KL-AA-10, suggesting that KL-AA-3.5 formed a relatively more rigid and compact adsorbed layer on the coated surface [49]. These results indicate that: (1) the higher charge density of KL-AA-10 enhanced its interaction with the polar oil phase (xylene), and (2) KL-AA-10 adopted more extended tail-and-loop conformations on the surface, likely entrapping greater amounts of water within the adsorbed layer [49]. Additionally, the physical entanglement of KL-AA-10 chains contributed to its rapid and substantial deposition on the surface (Figure 3). Notably, a decline in the deposited mass of KL-AA-10 was observed after approximately 250 s, suggesting partial detachment of loosely bound or physically entangled polymer chains from the adlayer. This behavior indicates that, while KL-AA-10 formed a thicker adsorption layer, it was comparatively less compact and structurally looser than that formed by KL-AA-3.5. It should be noted that the emulsions were prepared at concentrations above the CAC, where polymer aggregation may occur in the bulk phase and influence adsorption by promoting multilayer formation, aggregate adsorption, or altered interfacial packing. Therefore, the interfacial structure under emulsion conditions may differ from that measured by QCM-D. Also, in this study, the xylene-coated sensor was used as a model hydrophobic surface to mimic an oil-like interface, which did not directly represent liquid–liquid interfaces [18,50,51]. The QCM-D results reflect sub-CAC adsorption behavior and should therefore be interpreted as providing mechanistic insights into the relative adsorption behavior and interfacial layer characteristics of the polymers, rather than a direct measurement of the oil–water interface representing the exact interfacial structure under emulsification conditions.

### 3.5. Rheological Behavior of Xylene-Water Mixtures

**Figure 4:**
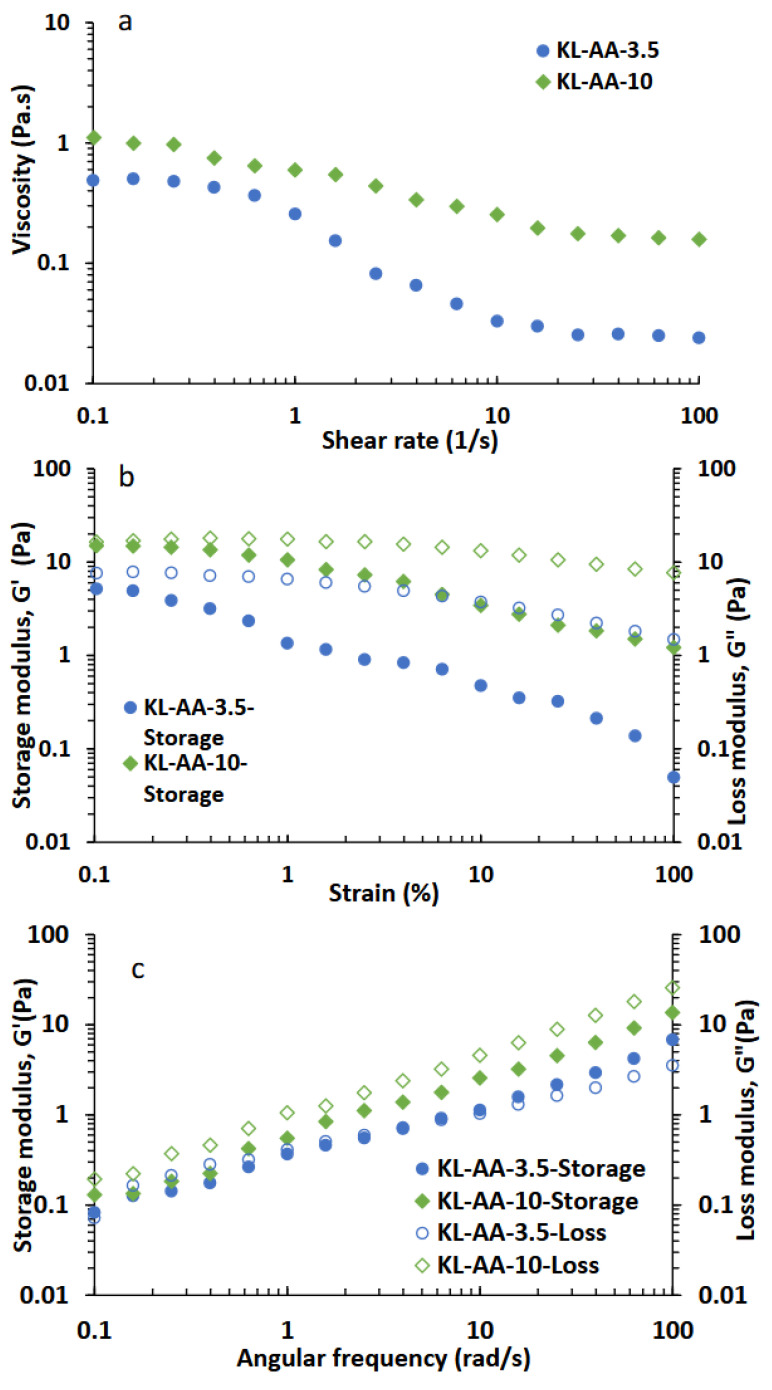
(**a**) Viscosity, (**b**) Storage/loss modulus behavior at a fixed frequency of 10 rad/s, and (**c**) Storage/loss modulus behavior as a function of angular frequency of KL-AA polymers at a 20 g/L in a water/xylene mixture.

**Figure 5:**
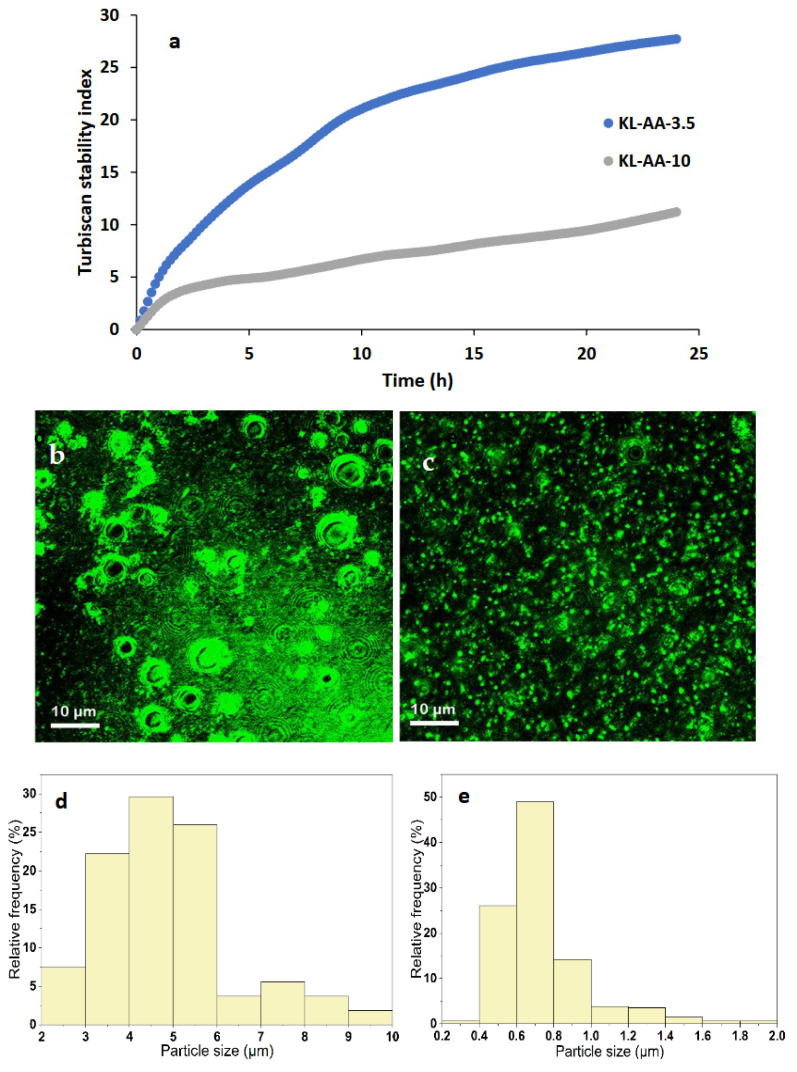
(**a**) The TSI value of xylene/water emulsion in the presence of KL-AA (20 g/L), Confocal images of the water/xylene emulsions prepared from xylene for (**b**) KL-AA-3.5 and (**c**) KL-AA-10. The oil droplet size distribution for water/xylene emulsions stabilized by (**d**) KL-AA-3.5 and (**e**) KL-AA-10.

The rheological behavior of the xylene–water emulsions is presented in Figure 4. As seen, all emulsions exhibited non-Newtonian flow behavior. Notably, clear differences in viscosity were observed among the samples. Variations in emulsion viscosity are generally influenced by interactions among molecules or particles within the continuous phase, as well as at the oil droplet interfaces [52]. Under applied shear stress, droplets may undergo deformation and fragmentation. When the applied stress is lower than the inter-droplet interaction forces, the emulsion exhibits elastic behavior, and energy is stored through deformation of the droplet network [53]. In a related study, Li et al. reported that ion–dipole interactions between carboxymethylated lignin molecules and kerosene droplets increased fluid viscosity [47]. The viscosity–shear rate data in Figure 4a were fitted using the Ostwald–de Waele (power-law) model. Both systems exhibited non-Newtonian shear-thinning behavior with flow behavior indices *n* < 1 (0.447 for KL-AA-3.5 and 0.682 for KL-AA-10). The higher consistency index (K = 0.56 Pa·s^n^) for KL-AA-10 emulsion compared with KL-AA-3.5 (K = 0.18 Pa·s^n^) corresponded to its more uniform droplet size distribution and improved interfacial stabilization, as shown in Figure 5, which collectively led to a stronger internal structure and higher resistance to flow. Consistent with these findings, KL-AA-10 produced emulsions with the highest viscosity across all shear rates, which could be attributed to its greater molecular weight and higher charge density (Table 1). These characteristics likely enhanced intermolecular interactions and strengthened the rheological structure of the xylene–water system compared to KL-AA-3.5.

To further elucidate emulsion behavior, strain amplitude sweep tests were conducted, and Figure 4b presents the storage modulus (G′) and loss modulus (G″) as functions of strain amplitude. The results indicate that the emulsions exhibited predominantly liquid-like characteristics (G″ > G′). At sufficiently large deformations, the internal structure of the emulsions was disrupted, leading to a rapid decline in G′ and an increasing dominance of viscous behavior. The observed elastic response of the emulsions can be attributed to strong hydrophobic interactions and electrostatic repulsion among emulsifier molecules at the droplet interfaces [54]. Notably, the KL-AA-10 stabilized emulsion exhibited consistently higher G′ and G″ values than the KL-AA-3.5 system, indicating a stronger internal structure and greater resistance to deformation. Frequency sweep measurements revealed different viscoelastic behaviors for the two systems (Figure 4c). For KL-AA-3.5, a crossover between storage modulus (G′) and loss modulus (G″) is observed at approximately 4 rad/s, indicating a transition from viscous-dominated behavior at low frequencies to elastic response at higher frequencies. This suggests the presence of a weak, transient network structure [55], which is in agreement with its broader droplet size distribution and less uniform interfacial structure (Figure 5). In contrast, the KL-AA-10 system exhibited G″ values consistently higher than G′ across the entire frequency range, indicating predominantly viscous behavior. However, both moduli were significantly higher than those of KL-AA-3.5, suggesting stronger intermolecular interactions and a more structured continuous phase. The higher viscoelastic moduli are likely associated with stronger interfacial films and enhanced droplet–droplet interactions in the KL-AA-10 system, which contributes to improved emulsion stability.

### 3.6. Stability Observation

The 24 h stability of the water–xylene emulsions (Figure 5a) demonstrates that KL-AA-10 provides significantly greater stability and a lower sedimentation rate than KL-AA-3.5. Although the more negative ζ-potential of KL-AA-10 may enhance electrostatic repulsion between droplets, ζ-potential alone does not govern emulsification performance. As both KL-AA samples reduce the interfacial tension of the xylene–water system to similar equilibrium values (Figure 2), interfacial tension alone is not a sufficient predictor of stability [56,57]. Instead, the improved stability arises from the combined effects of polymer conformation and viscoelastic properties. This suggests that while both polymers effectively saturate the interface, their adsorption configurations and viscoelastic contributions differ significantly [34]. KL-AA-10, owing to its higher grafting ratio, molecular weight, and charge density, likely forms a thicker and more extended interfacial layer, enhancing steric hindrance and electrostatic repulsion, and thereby reducing droplet coalescence even at comparable interfacial tension values [25].

Confocal microscopy images (Figure 5b,c) further reveal that KL-AA-10 produces smaller and more uniformly distributed xylene droplets than KL-AA-3.5. Quantitative image analysis (Figure 5d,e) shows that KL-AA-10 yields a substantially smaller droplet size distribution (D10 = 0.4 µm; D50 = 0.6 µm; D90 = 0.9 µm) compared to KL-AA-3.5 (D10 = 2.6 µm; D50 = 4.2 µm; D90 = 6.7 µm), indicating improved dispersion and reduced coalescence. Smaller droplets enhance kinetic stability by reducing sedimentation rates in accordance with Stokes’ law [12], while simultaneously increasing the total interfacial area that must be stabilized. The higher adsorption capacity of KL-AA-10 enables effective coverage of this expanded interface. Overall, the better emulsification performance of KL-AA-10 can be attributed to the synergistic effects of increased molecular weight and charge density, which promote electrostatic repulsion through ionized carboxylate (–COO^−^) groups, as well as steric stabilization arising from hydrated polymer chains at the interface. In addition, the formation of viscoelastic interfacial films, supported by QCM-D and rheological results (Figure 3 and Figure 4), together with the increased viscosity and viscoelastic moduli of the continuous phase (Figure 4), further suppress droplet collision and coalescence, collectively leading to enhanced emulsion stability.

### 3.7. Implication

The findings of this study demonstrate clear advantages over previously reported lignin-based emulsifiers, particularly sulfonated or oxidized lignins [12], which often require harsh reaction conditions or the use of toxic chemicals. In contrast, the lignin–acrylic acid copolymers developed here achieved enhanced adsorption at the oil interface, improved viscoelastic reinforcement of the continuous phase—key factors that collectively resulted in superior emulsion stability. Notably, the results highlight that optimizing molecular weight and charge density, rather than solely targeting interfacial tension reduction, is critical for improving emulsion performance. This insight has important implications for practical applications, suggesting that lignin-derived copolymers can serve as renewable, cost-effective emulsifiers for petroleum-related systems, including oil transport, enhanced oil recovery, drilling fluids, and industrial solvent emulsions, where long-term stability and rheological control are essential.

Although acrylic acid is derived from petroleum resources, the KL-AA copolymer contains a significant fraction of kraft lignin, which is a renewable byproduct of the pulp and paper industry. While this modification introduces a non-renewable component, the approach still increases the renewable content of the emulsifier compared with a conventional fully synthetic emulsifier. The biodegradability and environmental impact of the grafted lignin polymer remain important considerations and warrant further investigation in future studies. While the present study employs xylene as a model aromatic phase to enable controlled evaluation of interfacial behavior, future work will extend this approach to more complex and industrially relevant oil systems (e.g., crude oil or fuel mixtures) to further assess the practical applicability of KL-AA emulsifiers. Future work should focus on evaluating the performance of these polymers in more complex and realistic systems, such as high-salinity environments, elevated temperatures, and crude oil emulsions. Additionally, further studies are needed to investigate structure–function relationships, optimize synthesis scalability, and assess long-term environmental impacts and biodegradability to facilitate industrial implementation.

## 4. Conclusions

Overall, this work introduces a novel lignin-based emulsifier, lignin–acrylic acid copolymers (KL-AA), with increased molecular weight, charge density, and carboxylic acid contents, which enhance interfacial adsorption, electrostatic and steric stabilization, and overall emulsion stability compared to conventional lignin modifications with small functional groups. The results demonstrated that the lignin copolymer with higher molecular weight and charge density (KL-AA-10) exhibited greater adsorption onto the xylene surface compared to KL-AA-3.5. However, it formed a relatively looser adsorbed layer. Despite the differences in adsorption levels, these variations did not result in significant differences in the interfacial tension between xylene and water. Notably, the emulsion containing KL-AA-10 displayed higher viscosity and viscoelastic modulus values than the system stabilized with KL-AA-3.5, indicating that the greater deposition of KL-AA-10 contributed to strengthening the overall viscoelastic structure of the mixture. Furthermore, KL-AA-10 produced smaller xylene droplet sizes and enhanced viscoelastic properties, resulting in a xylene–water emulsion with improved stability and extended shelf life.

These findings suggest that (1) the water solubility of lignin is a critical factor in its effectiveness as an emulsifier in xylene–water systems, and (2) the molecular weight and charge density of lignin-derived polymers play key roles in their adsorption behavior at oil droplet interfaces, which ultimately governs the rheological properties and stability of the resulting emulsions.

This work demonstrates that grafting acrylic acid onto kraft lignin enhances its amphiphilicity and emulsifying performance relative to unmodified lignin. The results suggest that KL-AA copolymers are promising bio-based emulsifiers for applications requiring improved stability, such as coatings and multiphase formulations. More broadly, this study highlights the role of charge density and molecular weight in governing interfacial adsorption, film formation, and rheological behavior in lignin-based systems. Further studies on long-term stability, varying environmental conditions, and more complex, industrially relevant oil systems—along with benchmarking against conventional emulsifiers—are needed to assess their practical applicability fully.

## Figures and Tables

**Table 1:** Properties of KL and KL-AA.

**Sample**	**KL**	**KL-AA-3.5**	**KL-AA-10**
Lignin concentration, wt.%	4	4	4
Time, (h)	-	3	3
Temperature, (℃)	-	80	80
AA/lignin, (mol/mol)	-	3.5	10
Solubility, (g/L)	9.6	82.9	91.6
Charge density, (mmol/g)	0.1	1.1	4.7
M_w_, ×10^5^, (g/mol)	2.13	3.07	7.99
M_n_, ×10^5^, (g/mol)	1.46	1.52	2.04
M_w_/M_n_,	1.58	2.0	3.9
Carboxylate group, (mmol/g)	0.17	1.09	3.23
Phenolic hydroxyl group, (mmol/g)	3.26	1.57	0.84
Carbon, (wt.%)	64	60	50
Hydrogen, (wt.%)	7.0	6.4	5.8
Oxygen (wt.%)	27	35	44
Zeta potential in solution, (-mV)	-	38	52

## Data Availability

The original contributions presented in this study are included in the article. Further inquiries can be directed to the corresponding authors.
